# Commentary: Exploring decision-making in home care nursing

**DOI:** 10.1177/17449871251362922

**Published:** 2025-10-30

**Authors:** Mubarak Patel

**Affiliations:** Research Fellow, The Birmingham Centre for Evidence and Implementation Science, UK

**Commentary to:** Nurses’ perceptions of shared decision-making with older adults using home care services in South Korea: a cross-sectional study (Doan et al., 2026).

This cross-sectional study investigates how home care nurses in South Korea perceive their involvement in shared decision-making (SDM) with older adults. A total of 154 nurses completed an online survey in August to September 2023, collecting socio-demographic data and the nurses’ self-perceived levels of SDM involvement using a five-item questionnaire in the context of home care.

This questionnaire included 15 questions, covering the following aspects:

- Informing clients about the option to choose treatment or a care plan
- Presenting the advantages and disadvantages of available options
- Inquiring about clients’ preference for care
- Discussing preferred options
- Involving clients in the care plan to the extent they wished

The analysis employed descriptive statistics, analysis of variance (ANOVA), Pearson correlations, and multiple linear regression to identify prediction of nurses’ self-reported SDM involvement.

Key findings showed that nurses felt moderately involved in SDM (mean score of 3.73 out of 5) but less involved than family caregivers, which included spouses and children. The most difficult decision reported for older adults was whether to remain at home or move into assisted living facilities. The regression analysis found that the factors that predicted higher SDM for nurses were longer duration of home care experience, more frequent decision-making opportunities, and working in long-term care settings; with field of work showing the largest coefficient in the model, suggesting that it is one of the larger influential factors.

From a statistical viewpoint, the findings align with expectations in the field: more experienced nurses and those more involved in decision-making are more confident in SDM and participate at a higher rate, compared to those with less experience. However, the modelling does suggest that there are unmeasured factors that may influence SDM engagement, for example perhaps personal attitudes to SDM, which highlights a need for future research in this area.

The findings support theories of experiential learning and person-centred care and, while the association between experience and SDM was modest in the regression model, structured support and training likely enhance this effect.

Key messages from this paper that can be taken forward include the lack of formal SDM training among the responding nurses with only 9.1% receiving any formal training, underlining a curricular gap. It also highlights that policies should be put in place which focus on training nurses in SDM where SDM involvement was low, such as community or public health centres. SDM involvement increases with exposure and frequency, suggesting that more experienced nurses should take a proactive approach in mentoring less experienced nurses; and recognising the central role of close family members, which can lead to better and more inclusive care planning which supports all relevant participants.

Overall, this study is statistically sound and robust, using validated tools and conducting the appropriate statistical tests. However, the use of self-reported data and the modest model fit imply more research is needed. Despite this, the study offers meaningful insights and lays a good foundation for nurse-led intervention and system-level planning.
